# Update to state of the Journal of Patient-Reported Outcomes

**DOI:** 10.1186/s41687-024-00715-x

**Published:** 2024-06-12

**Authors:** Rasa Ruseckaite, Chih-Hung Chang

**Affiliations:** 1 School of Public Health and Preventive Medicine, Monash University, Melbourne, Australia; 2 Washington University School of Medicine, St. Louis, USA

Almost eight years ago, in 2016, the International Society for Quality of Life Research (ISOQOL) Board established the Journal of Patient-Reported Outcomes (JPRO). The JPRO is an international, open access, multi-disciplinary journal publishing original manuscripts in the field of patient-reported outcomes (PROs) and patient-centered outcomes (PCO) research. Thanks to the hard work of the Editorial teams over the years, the JPRO received its first impact factor of 2.7 in 2022 with more than 400,000 annual article downloads.

Since the inception of the JPRO we have witnessed a continued rise in the number of submissions, reflecting the growing global interest in PROs. We receive approximately 300 submissions annually, with nearly half of them being published. The disparity between what is submitted and what gets published is driven by scope and the scientific quality of submissions. In this editorial, we aim to address both of these issues.

The Journal invites high quality manuscripts covering a broad range of topics in PROs and PCO. We consider original research and review articles, brief communications, commentaries, editorials, and reviews of recent books and software advances in the following areas: (1) PROs in clinical trials; (2) PROs in clinical practice; (3) Patient and public involvement and engagement in PROs research; (4) studies on the development and application of PROs; and (5) social and behavioural determinants of health and PRO measures. In general, the JPRO does not publish case reports, articles reporting study designs, pilot studies, or feasibility studies.

We encourage submissions reporting Patient-Reported Experience Measures (PREMs) and measures of patient satisfaction. Manuscripts describing innovative methods of capturing and analysing patient or caregiver reports of their experiences receiving care, as well as analyses of factors that may impact experiences and satisfaction, are welcome.

The JPRO uses the *ISOQOL Dictionary of Quality of Life and Health Outcomes Measurement* definitions of PROs, PROMs (Patient-Reported Outcomes Measures) and PREMs [1]. All manuscripts must be written in English and meet professional standards for grammatical accuracy, quality and style. All references mentioned in the Reference List should be cited in the text, and vice versa. Ethics and consent process must be stated in the manuscript. All submissions must provide strong rationale and articulate implications of the study. Crucially, we expect methods employed to be an appropriate fit to the research question(s) being addressed. Manuscripts should include sample size calculations, list all data exclusions, describe all manipulations and all measures used in the study. Qualitative studies should clearly describe the methodological approach to ensure that standards for methodological quality are met. Studies that use a mixed method approach should meet the requirements for qualitative and quantitative methodologies. The results should describe all the experiments and calculations performed and the findings observed. The discussion should describe conclusions drawn from the results, as well as the significance and implications of the research.

All submissions are checked by the Editors to determine whether the subject and content are appropriate for the journal. Manuscripts that enter the review process are assigned to an Editor with expertise in the subject area. Reviewers are recruited and they are expected to submit their assessment within three weeks. The Editor then makes an editorial decision that is sent to the corresponding author. In 2023, the average time to the first decision for manuscripts sent out for peer review was 54 days, with the average time to the final decision being approximately 117 days.

The JPRO Editorial Team is composed of internationally renowned Editors with unique and complementary expertise in PROs. Serving as Co-Editors-in Chief are Dr. Rasa Ruseckaite (Monash University, Australia) and Dr. Elizabeth J. Unni (Touro College of Pharmacy). Additionally, we have five Associate Editors on the team: Dr. Jill Carlton (University of Sheffield, UK), Dr. Kate Williams (Acaster Lloyd Consulting Ltd, UK), Linda M. Nelsen (GSK, USA), Dr. Elizabeth Marfeo (Tufts University, USA), Dr. John Devin Peipert (Northwestern University, USA), and Dr. Jessica Roydhouse (University of Tasmania, Australia). Our International Editorial Advisory Board currently includes 24 internationally recognized researchers; we warmly thank them for their commitment and contribution to the journal. We also thank the ISOQOL Board of Directors, all the reviewers, researchers, clinicians and patients for their continuous support of the Journal.
